# Gastric hamartomatous inverted polyp: Report of three cases with a review of the endoscopic and clinicopathological features

**DOI:** 10.1002/deo2.198

**Published:** 2023-01-04

**Authors:** Takuya Ohtsu, Yu Takahashi, Mitsuo Tokuhara, Tomomitsu Tahara, Mitsuaki Ishida, Chika Miyasaka, Koji Tsuta, Makoto Naganuma

**Affiliations:** ^1^ Division of Gastroenterology and Hepatology, Third Department of Internal Medicine Kansai Medical University Osaka Japan; ^2^ Department of Pathology and Division of Diagnostic Pathology Kansai Medical University Osaka Japan; ^3^ JCHO Hoshigaoka Medical Center Gastroenterology and Hepatology Osaka Japan

**Keywords:** endoscopic ultrasound, gastric hamartomatous inverted polyp, gastric neoplasm, pathology, submucosal tumor

## Abstract

**Objectives:**

A gastric hamartomatous inverted polyp (GHIP) is a rare submucosal tumor characterized histopathologically by a submucosal inverted proliferation of cystically dilated hyperplastic gastric glands. Only 42 GHIPs have been reported in English literature. Few GHIPs have been reported to accompany adenocarcinomas. We reported on three patients with a GHIP and reviewed the clinicopathological and endoscopic features of GHIPs.

**Methods:**

This study included two men and one woman with a GHIP. The endoscopic, histopathological, and immunohistochemical features of the endoscopically resected specimens were analyzed. A gene mutation analysis was also performed.

**Results:**

All the tumors were located in the body of the stomach, with a median size of 20 mm. Two tumors were sessile, and the remaining tumor had a pedunculated appearance. The overlying mucosa mainly appeared normal but was reddish in one tumor. The histopathological examination of the tumors revealed a well‐circumscribed and lobular submucosal proliferation of cystically dilated hyperplastic glands. The immunohistochemical analysis revealed that the MUC5AC‐positive foveolar epithelium was located in the center, and MUC6‐positive pseudo‐pyloric or pepsinogen‐I and H^+^/K^+^ ATPase‐positive fundic‐type glands were located at the periphery of two tumors. No carcinomatous components were noted in any of the tumors. Moreover, no significant mutations in oncogenes or tumor suppressor genes were noted.

**Conclusions:**

Our review revealed that approximately three fourths of GHIP cases showed an submucosal tumor‐like feature, whereas endoscopic features, including the endoscopic ultrasonography findings, were not characteristic. Because an endoscopic diagnosis of a GHIP may be difficult, complete endoscopic resection may be required for a pathological diagnosis.

## INTRODUCTION

A gastric hamartomatous inverted polyp (GHIP) is an extremely rare tumor characterized histopathologically by a submucosal inverted proliferation of cystically dilated hyperplastic gastric glands accompanying a branching proliferation of smooth muscle bundles.^1^ GHIP has also been referred to as an “inverted hyperplastic polyp,” and collectively, lesions showing inverted growth are called “gastric inverted polyps.”^1^ Due to the rarity of the tumor, a GHIP is difficult to diagnose based on endoscopic findings. Moreover, the endoscopic and clinicopathological features, as well as the pathogenesis of GHIPs, are poorly understood. In addition, few patients with a GHIP accompanying an adenocarcinoma have been reported^6^, ^8^, ^11^, ^15^, ^27^; thus, it has been suspected that GHIPs may have precancerous potential. However, a comprehensive gene analysis of GHIPs has not yet been performed. In this study, we reported on the endoscopic and clinicopathological features of three cases of GHIPs and reviewed their clinicopathological features. In addition, we aimed to perform a gene mutation analysis of the GHIPs, for the first time, and to discuss the malignant potential of GHIPs.

## MATERIALS AND METHODS

### Case selection

This was a retrospective review of endoscopically resected specimens diagnosed as “hamartomatous polyps” or “hamartomatous inverted polyps” of the stomach at our hospital from January 2006 to November 2020, and we re‐diagnosed three lesions as GHIPs. The definition of GHIP was based on previous reports: a heterotopic inverted proliferation of cystically dilated hyperplastic gastric glands in the submucosal layer accompanying a proliferation of muscular bundles.^1^, ^4^, ^7^ None of the patients had Peutz–Jeghers syndrome or any other polyposis syndrome.

This retrospective, single‐institution study was conducted in accordance with the principles of the Declaration of Helsinki, and the study protocol was approved by the institutional review board of the Kansai Medical University Hospital (Approval #2020255). Due to the retrospective nature of the study, informed consent was obtained from the patients using the opt‐out methodology, with no risk to the participants. Information with respect to this study, such as the inclusion criteria and the opportunity to opt out, was provided via the institutional website.

### Endoscopic findings

An esophagogastroduodenoscopy (Olympus GIF‐Q260, GIF‐H260Z, GIF‐H290Z) and an endoscopic ultrasonography (EUS, Olympus UM‐2R [12 MHz], Patients 1 and 2; Olympus UM‐3R [20 MHz], Patient 3) were performed on all the patients. The location, macroscopic features (sessile or pedunculated), features of the surface of the lesions, and the presence of accompanying lesions were analyzed using the esophagogastroduodenoscopy findings, and the internal nature of the lesions using the EUS findings. A polypectomy, an endoscopic mucosal resection, and an endoscopic submucosal dissection were performed for each patient.

### Histological evaluation and immunohistochemistry

For the histological examination, the endoscopically resected specimens were fixed in 10% buffered formalin and embedded in paraffin. The tissues were sectioned and stained with hematoxylin and eosin. The histopathological findings, including the glandular components (foveolar, fundic, cardiac, pyloric, intestinal, or acinic types), nuclear atypia, and the characteristics of stroma, muscularis mucosae, and the surrounding mucosa, were analyzed histopathologically.

Immunohistochemical analyses were performed using an autostainer (Discovery ULTRA System; Roche Diagnostics, Basel, Switzerland), according to the manufacturer's instructions. The primary antibodies used in the present study were a rabbit monoclonal antibody against CDX2 (EPR2764Y, pre‐diluted, Roche), a mouse monoclonal antibody against H^+^/K^+^ ATPase (1H9, dilution 1:1000; MBL, Nagoya, Japan), a mouse monoclonal antibody against Ki‐67 (MIB1, dilution 1:100; Agilent, Santa Clara, CA, USA), a mouse monoclonal antibody against MUC2 (MRQ‐18, pre‐diluted, Roche), a mouse monoclonal antibody against MUC5AC (MRQ‐19, pre‐diluted; Roche), a mouse monoclonal antibody against MUC6 (MRQ‐20, pre‐diluted; Roche), and a mouse monoclonal antibody against pepsinogen‐I (8003 [99/12], dilution 1:100; Bio‐Rad Laboratories, Hercules, CA, USA). Two researchers conducted an independent evaluation of the immunohistochemical staining results.

### Mutational analysis

A genetic analysis using the AmpliSeq Cancer Hotspot Panel (Illumina), which can detect hot‐spot regions of approximately 2800 mutations from 50 oncogenes and tumor suppressor genes, was performed for all three lesions.

## RESULTS

### Clinical and endoscopic findings

**FIGURE 1:**
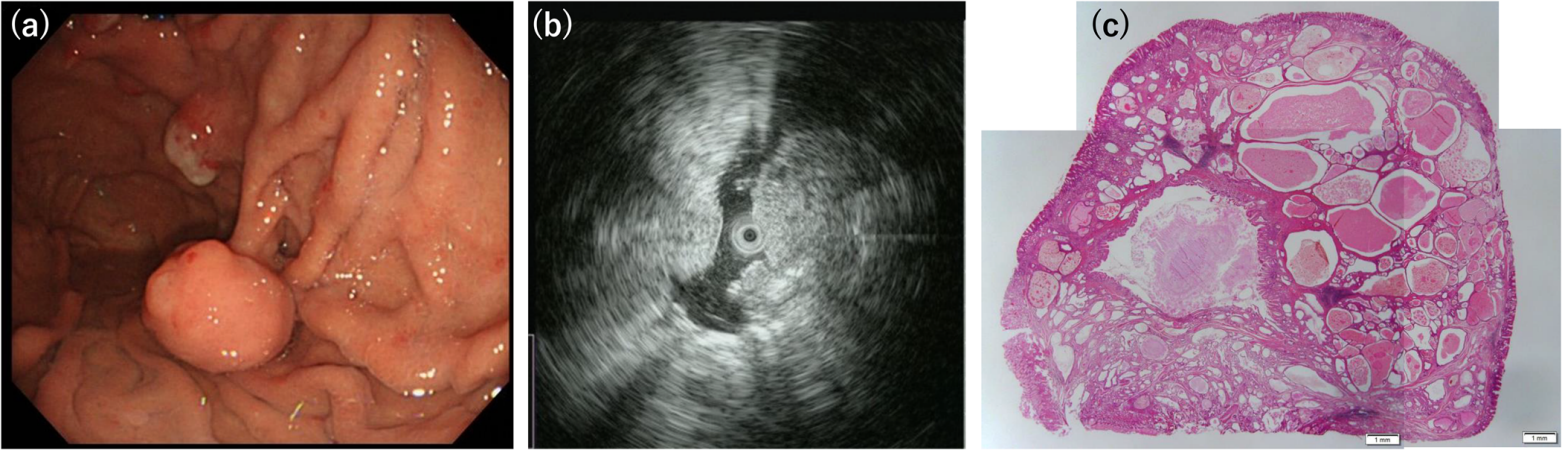
Endoscopic and histopathological features of Patient 1. (a) A pedunculated submucosal tumor is noted, and the surface is covered with normal mucosa. (b) Endoscopic ultrasonography shows that the tumor has a high echoic mass with a low echoic spot. (c) Submucosal proliferation of cystically dilated hyperplastic glands and the surrounding smooth muscle, covered by intact mucosa (hematoxylin and eosin staining, panoramic view)

Table 1 summarizes the clinical and endoscopic findings of the study. This study included two men and one woman, whose ages at diagnosis ranged from 62 to 69 years (median, 67 years). Two patients had no clinical signs or symptoms, whereas one presented with hematemesis and anemia (Patient 1). In one patient, esophageal cancer coexisted (Patient 2). All the tumors were located in the body of the stomach. The median tumor size was 20 mm (range 18–21 mm). Two tumors were sessile, and the remaining tumor (Patient 1) had a pedunculated appearance on endoscopic examination. Endoscopically, the overlying mucosa was mainly intact and normal in two patients and reddish in one patient (Patient 2) (Figures 1a, 3a, and 5a). The assessment of the background mucosa demonstrated that all the patients had atrophic gastritis. The EUS revealed two tumors that appeared as heterogeneous lesions containing small cystic areas (Patients 1 and 2; Figures 1b and 3b). In the remaining tumor, the liquid components were stored in the first layer, and the solid components were present inside (Patient 3; Figure 5b). All the patients were discharged without any complications after the endoscopic procedures.

**TABLE 1:** Clinical and endoscopic features of three patients with gastric hamartomatous inverted polyp

						Endoscopic findings			
Patient no.	Age	Sex	Location	Size (mm)	Procedure	Appearance	Features of the surface	Aperture to the surface	Past history
1	69	Male	Upper body	20 × 20 × 15	Polypectomy	Pedunculated	Ruggedness	×	Mitral, aortic, tricuspid valves replacement, atrial fibrillation, chronic hepatitis C, liver cirrhosis, cholelithiasis, diabetes
2	69	Male	Lower body	18 × 15 × 15	EMR	Sessile	Ruggedness and reddish	×	Hypertension, dyslipidemia, squamous cell carcinoma of the esophagus, arteriosclerosis obliterans
3	62	Female	Middle body	21 × 16 × 14	ESD	Sessile	Surface smoothing	×	Ovarian cyst, uterine fibroid, hyperlipidemia

Abbreviations: EMR, endoscopic mucosal resection; ESD, endoscopic submucosal dissection.

### Histopathological examination

The histopathological examination revealed a well‐circumscribed and lobulated submucosal proliferation of cystically dilated hyperplastic glands that covered the entire mucosa (Figures 1, 3, and 5c). No obvious continuity between the submucosal glands and the surface mucosa was observed in the three tumors. The glandular components of the submucosal proliferation included a pseudo‐pyloric and fundic‐type and a foveolar epithelium. In two tumors (Patients 1 and 2), the gastric mucosal architecture was maintained in the cystically dilated glands in the submucosa because the pseudo‐pyloric and fundic‐type glands were located at the periphery of the cystically dilated glands, and the hyperplastic foveolar epithelium was present in the center of the dilated glands (Figures 1c and 3c). No nuclear atypia was observed in the glandular cells. Smooth muscle bundles were observed surrounding the lobulated dilated glands and/or outside the lesion. The superficial mucosa was covered with a normal fundic gland‐type mucosa in all the tumors, and intestinal metaplasia was noted in one tumor (Patient 3). No malignant lesions were observed in any of the patients.

### Immunohistochemical staining

**FIGURE 2:**
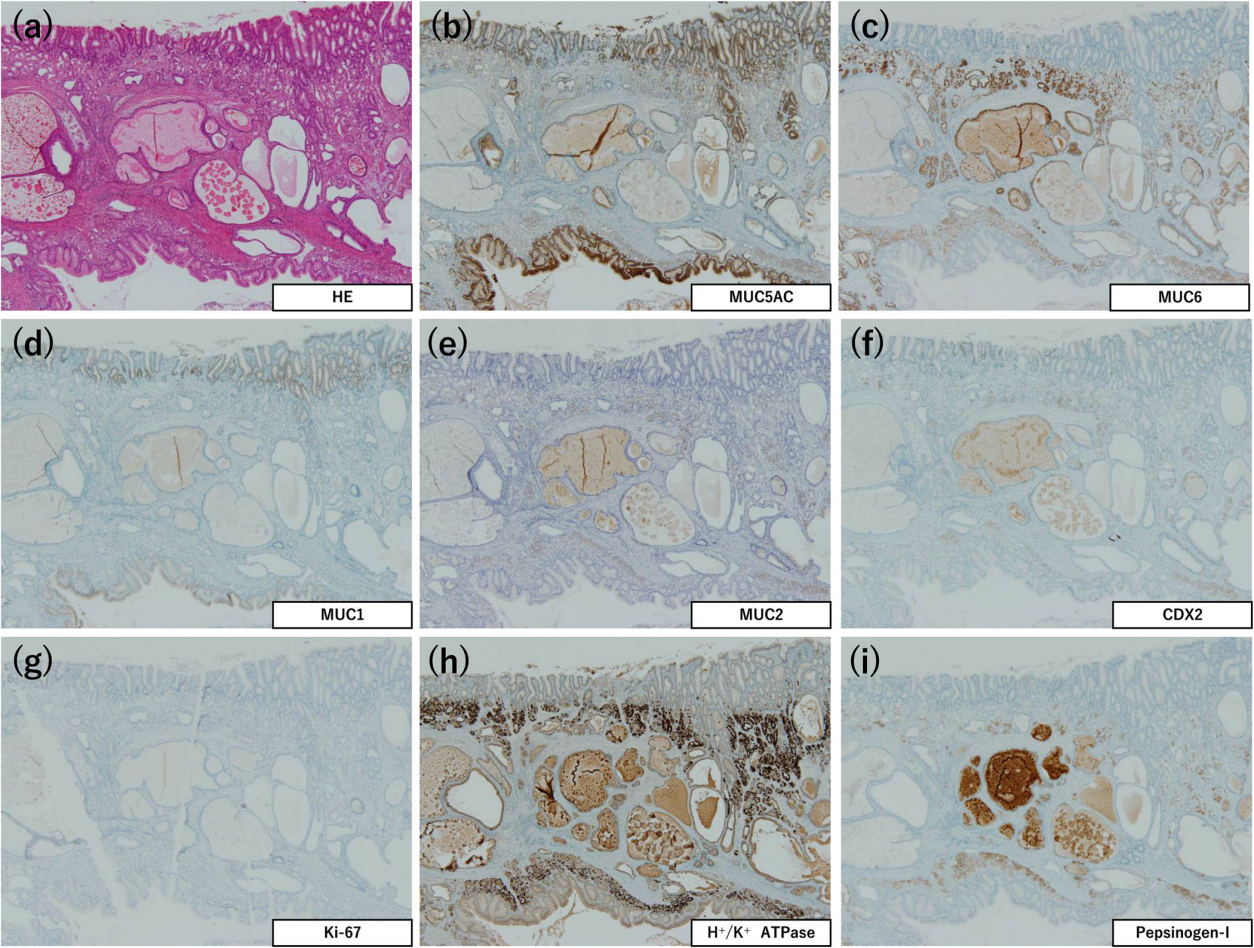
Immunohistochemical features of Patient 1. (a) Hematoxylin and eosin staining. (b) MUC5AC is expressed mainly at the center of the dilated glands. (c) MUC6 is expressed mainly at the periphery of the dilated glands. (d) MUC1 is focally expressed. (e) MUC2 is not expressed. (f) CDX2 is focally expressed. (g) Ki‐67 labeling index is less than 1%. (h) H^+^/K^+^ ATPase is expressed. (i) Pepsinogen‐I is expressed. (×40 magnification (a)–(i))

The immunostaining results are summarized in Table 2. This revealed that MU5AC expression was observed mainly at the center of the dilated glands (Figures 2b, 4b, and 6b). MUC6 and pepsinogen‐I were expressed mainly at the periphery of the dilated glands (Figures 2, 4, 6c,i). H^+^/K^+^ ATPase was expressed in the fundic‐type glands of one tumor (Patient 1; Figure 2h), and a few H^+^/K^+^ ATPase‐positive cells were also observed in the remaining two tumors (Patients 2 and 3; Figures 4h and 6h). No MUC2‐positive cells were found in any of the tumors, whereas a few CDX2‐positive cells were noted in all three tumors (Figures 2, 4, and 6f). The Ki‐67 labeling index was less than 2% in all three tumors (Figures 2, 4, and 6g).

**TABLE 2:** Immunohistochemical results of three patients with gastric hamartomatous inverted polyp

	Immunohistochemical stainings							
Patient no.	MUC1	MUC2	MUC5AC	MUC6	CDX2	Ki‐67 labeling index	Pepsinogen‐I	H^+^/K^+^ ATPase
1	△	×	〇	〇	△	<１％	〇	〇
2	△	×	〇	〇	△	<2％	〇	△
3	×	×	〇	〇	△	2%–3％	〇	△

*Note*: 〇, positive; △, focal positive; ×, negative.

**FIGURE 3:**
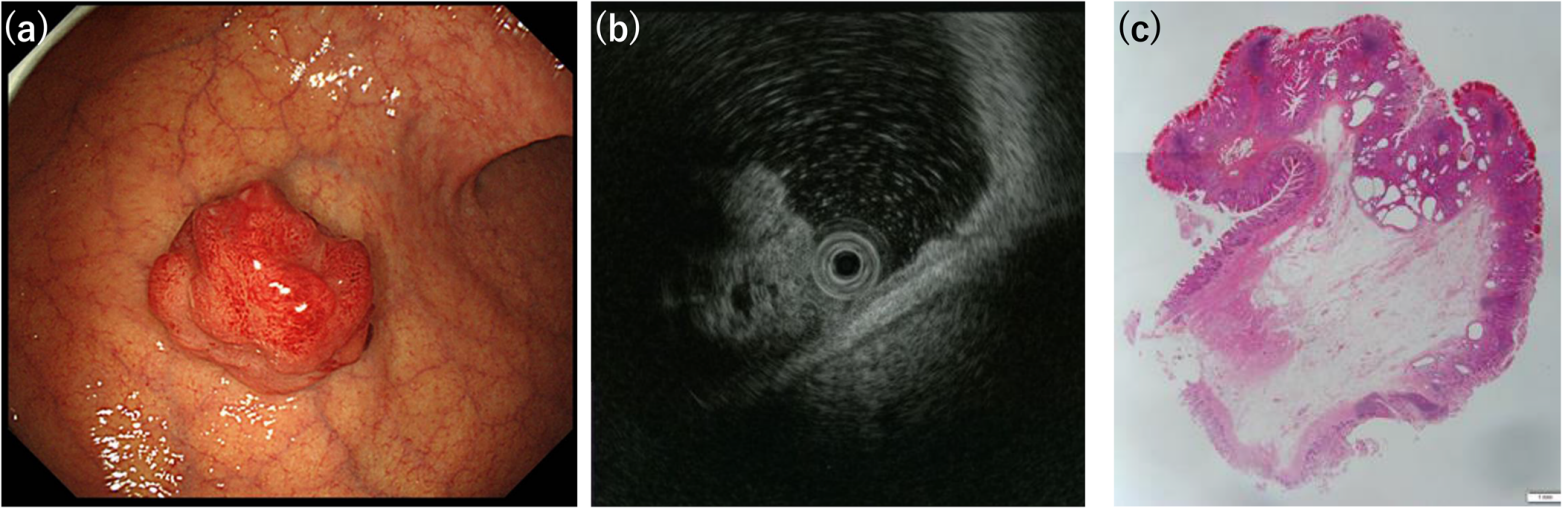
Endoscopic and histopathological features of Patient 2. (a) A sessile submucosal tumor is present, and the surface is covered with reddish mucosa. (b) Endoscopic ultrasonography finding. The tumor has a hyperechoic mass with a small anechoic spot. (c) A well‐circumscribed and lobulated submucosal proliferation of cystically dilated hyperplastic glands is covered by intact mucosa (hematoxylin and eosin staining, panoramic view)

**FIGURE 4:**
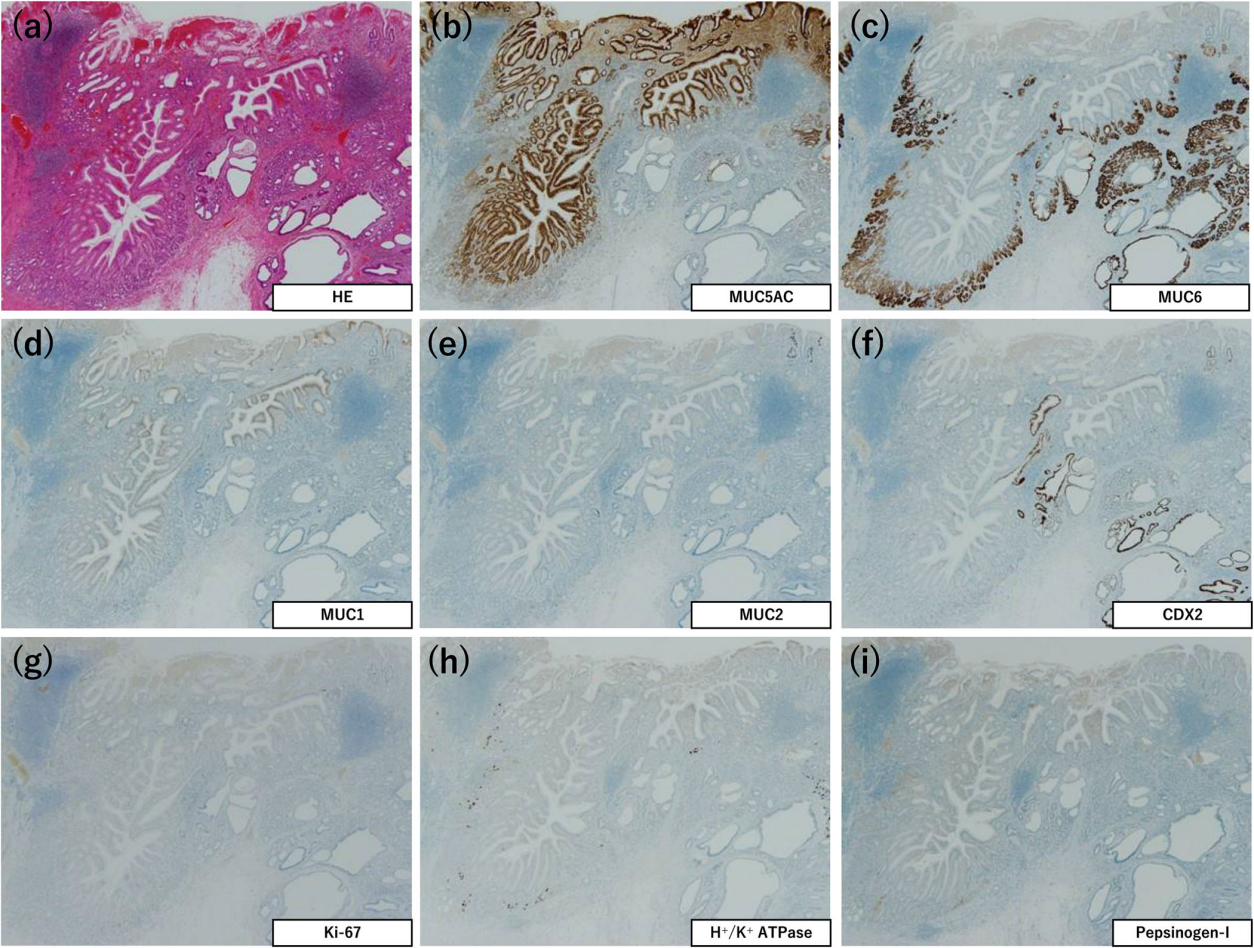
Immunohistochemical features of Patient 2. (a) Hematoxylin and eosin staining of the tumor. (b) MUC5AC is expressed mainly at the center of the dilated glands. (c) MUC6 is expressed mainly at the periphery of the dilated glands. (d) MUC1 is expressed focally. (e) MUC2 is not expressed. (f) CDX2 is focally expressed. (g) Ki‐67 labeling index is approximately 2%. (h) H^+^/K^+^ ATPase is focally expressed. (i) Pepsinogen‐I is focally expressed. (×40 magnification (a)–(i))

**FIGURE 5:**
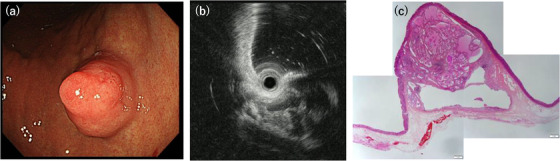
Endoscopic and histopathological features of Patient 3. (a) A sessile submucosa tumor is noted. The surface is smooth and is covered with normal mucosa. (b) Endoscopic ultrasonography shows anechoic components in the submucosal layer with partition walls and hyperechoic mass. (c) A well‐circumscribed and lobulated submucosal proliferation of cystically dilated hyperplastic glands. No mucosal communication is noted (hematoxylin and eosin staining, panoramic view)

**FIGURE 6:**
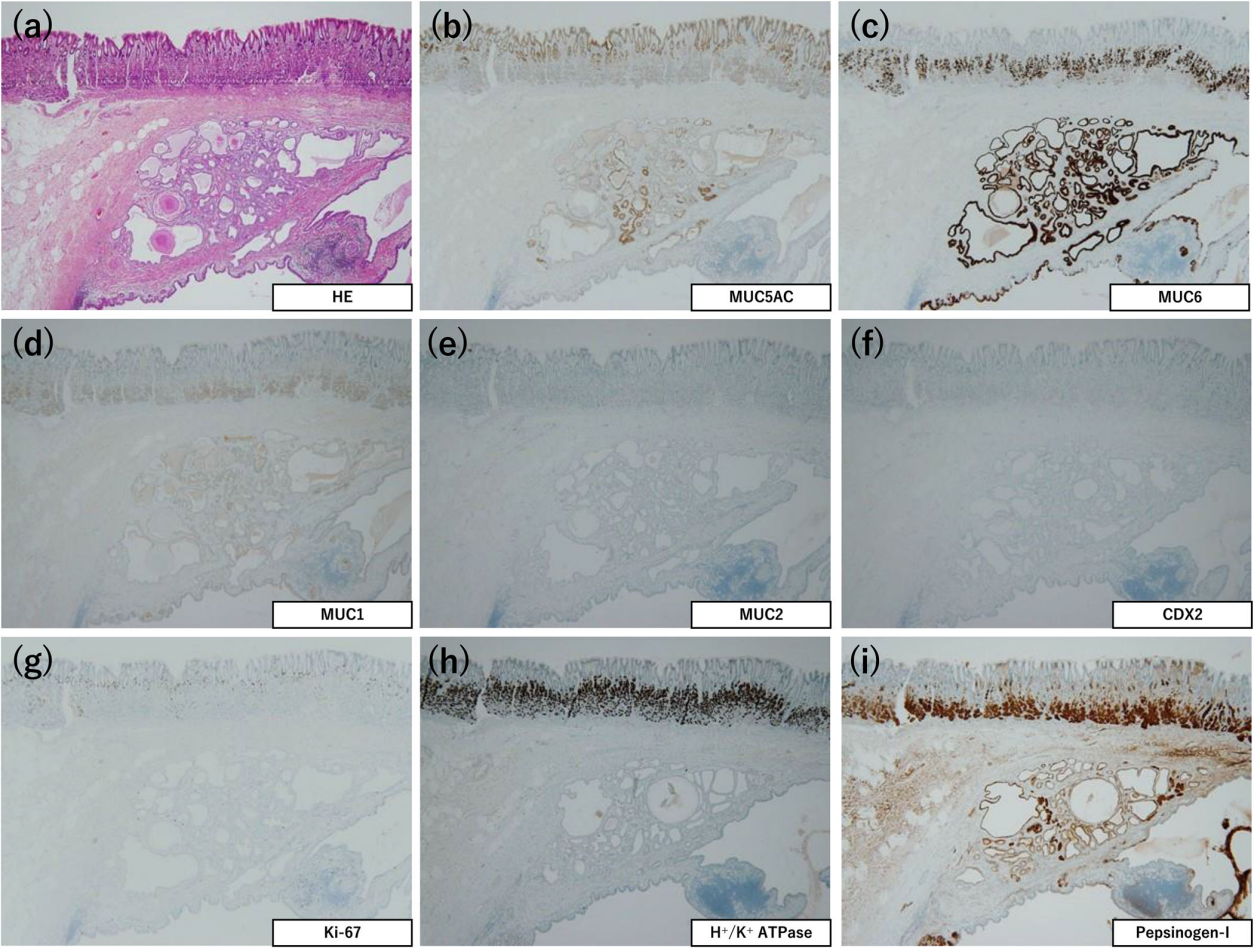
Immunohistochemical features of Patient 3. (a) Hematoxylin and eosin staining of the tumor. (b) MUC5AC is expressed mainly at the center of the dilated glands. (c) MUC6 is expressed mainly at the periphery of the dilated glands. (d) MUC1 is not expressed. (e) MUC2 is not expressed. (f) CDX2 is expressed focally. (g) Ki‐67 labeling index is approximately 2%. (h) H^+^/K^+^ ATPase is expressed focally. (i) Pepsinogen‐I is expressed. (×40 magnification (a)–(i))

### Gene mutation analysis

No significant mutations in the oncogenes and tumor suppressor genes, including *p53*, *KRAS*, and *GNAS*, were observed in any of the tumors.

## DISCUSSION

In the present study, we reported the clinicopathological and endoscopic features of three patients with GHIPs (the third largest case study of GHIPs) and analyzed the genetic alteration in GHIPs, for the first time.

Various polypoid lesions can occur in the stomach. GHIP is an extremely rare type of gastric polyp, and various terms, including inverted hyperplastic polyps, have been coined for this lesion. To the best of our knowledge, only 41 previously reported patients with GHIP have been reported in the English‐language literature (41 patients, 42 tumors because 1 patient had 2 GHIPs^6^).^2^, ^3^, ^4^, ^5^, ^6^, ^7^, ^8^, ^9^, ^10^, ^11^, ^12^, ^13^, ^14^, ^15^, ^16^, ^17^, ^18^, ^19^, ^20^, ^21^, ^22^, ^23^, ^24^, ^25^, ^26^, ^27^ Only 9 patients with GHIPs, 24 patients with inverted hyperplastic polyps (1 patient had 2 lesions), and 8 patients with similar lesions given other names have been reported. Table 3 summarizes the clinicopathological and endoscopic features of these patients (Table S3 shows the detailed clinicopathological and endoscopic characteristics of the 42 tumors). Our review of these features of the previously reported patients, as well as the present 3 patients (44 patients, 45 tumors), revealed that there was a slight male predominance (male:female 25:19), and the median age of the patients was 58 years (range, 23–81 years). Most of the tumors were asymptomatic and incidentally found; however, some patients presented with epigastric pain or discomfort,^5^, ^11^ fatigue and nausea secondary to obstruction due to the tumor,^24^ or anemia secondary to chronic hemorrhage.^7^ The most frequent location of the GHIPs was in the body (32 tumors), followed by the fundus (8 tumors), antrum (3 tumors), and cardia (2 tumors) (Table 3). The median tumor size was 15 mm (range, 3.5–45 mm). Differentiating a GHIP from the submucosal heterotopic gastric mucosa may be difficult. A submucosal heterotopic gastric mucosa is not a rare lesion. A study from Japan reported the presence of this lesion in 10.7% of the resected stomach specimens,^28^ and their histopathological features might overlap. However, GHIP is referred to commonly as a relatively large solitary elevated lesion and is characterized histopathologically by the proliferation of muscular bundles.^1^, ^4^, ^7^ Additional clinicopathological studies are needed to clarify the classification of these submucosal proliferative lesions.

**TABLE 3:** Summary of the clinicopathological and endoscopic features of the previously reported gastric hamartomatous inverted polyp

	Submucosal tumor type (*n* = 34)	Polyp type (*n* = 10)
Male/female	21/13	4/6
Age (years)	55	64
	(23–81)	(34–70)
Size (mm)	18	14
	(3.5–30)	(10–45)
Location	Cardia: 2; fundus: 3; body: 25; antrum: 2	Cardia: 0; fundus: 3; body: 6; antrum: 1
Accompanied lesion	Normal: 5; intestinal metaplasia: 6; gastritis cystica profunda: 2; chronic gastritis: 1; atrophic gastritis: 7; *Helicobacter pylori*—associated gastritis: 2; nonspecific gastritis: 2; not available: 13	Normal: 4; intestinal metaplasia: 1; gastritis cystica profunda: 0; chronic gastritis: 1; atrophic gastritis: 2; *Helicobacter pylori*—associated gastritis: 1; nonspecific gastritis: 0; not available: 2
Coexisting carcinoma	5	1
Treatment	Gastrectomy: 7; wedge resection: 3; endoscopic mucosal resection: 3; endoscopic submucosal dissection: 10; laparoscopy and endoscopy and cooperative surgery, 1; modified CLEAN‐NET, 1; not available 9	Wedge resection: 1; polypectomy: 3; endoscopic mucosal resection (EMR): 1; endoscopic submucosal dissection (ESD): 2; not available: 3

The endoscopic features of GHIPs are diverse. Aoki *et al.* classified the endoscopic features of GHIPs into submucosal tumors (SMTs) and polyps.^7^ The SMT type does not have a stalk, whereas the polyp type has a stalk.^7^ According to their classification, the SMT type was more frequently noted in a GHIP (SMT type: 34 tumors and polyp type: 10 tumors) (Tables 1 and 3). Endoscopically, the surface of the lesion was typically covered with normal mucosa, and an erosion or depression was occasionally noted. An opening from the center to the surface with mucus outflow was observed (15 tumors) (the relationship between this finding and the pathological features is discussed below). The assessment of the background mucosa revealed the presence of normal mucosa (12 patients), atrophic gastritis (10 patients), intestinal metaplasia (8 patients), *Helicobacter pylori*‐associated gastritis (3 patients), gastritis cystica profunda (2 patients), nonspecific gastritis (2 patients), chronic gastritis (1 patient), and gastritis (details unknown, 4 patients).

An EUS has been routinely performed for the qualitative diagnosis of SMT or to determine the depth of invasion of gastric tumors and has often revealed the presence of a multilocular anechoic region in the second or third layer of the gastric wall of the GHIP.^7^, ^8^, ^9^, ^10^, ^11^, ^12^, ^13^, ^14^, ^15^, ^16^, ^17^, ^18^, ^19^, ^20^, ^21^, ^22^, ^23^, ^24^, ^25^, ^26^, ^27^ However, no specific features of GHIPs have been identified, and in the present series, the EUS revealed an atypical image of a cyst with partition walls and a hyperechoic nodule. In addition, previous reports showed that an EUS was performed in 25 patients (including six patients with adenocarcinoma components); however, no significant differences were noted in the EUS features of a GHIP with or without adenocarcinoma.^7^, ^8^, ^9^, ^10^, ^11^, ^12^, ^13^, ^14^, ^15^, ^16^, ^17^, ^18^, ^19^, ^20^, ^21^, ^22^, ^23^, ^24^, ^25^, ^26^, ^27^ Therefore, while an endoscopic diagnosis of a GHIP using the findings of EUS alone may not be possible, the EUS may be useful in determining whether an endoscopic resection may be performed. In the present study, only three patients had all the tumors endoscopically resected because one patient presented with hematemesis and anemia (Patient 1). Although the other patients experienced no symptoms (Patients 2 and 3), an endoscopic resection was performed for the pathological diagnosis because of the relatively large size of the tumors.

Recently, Kim *et al.* reported the largest series of GHIP cases and classified this tumor into three types based on the histopathological features.^27^ Type 1 characteristically has a communicating structure with an overlying mucosa and a well‐defined smooth muscle boundary showing a normal gastric mucosal architecture (a hyperplastic foveolar epithelium at the center of the submucosal cysts and pyloric or fundic glands at the periphery). Type 2 is characterized by an isolated submucosal lesion surrounded by smooth muscle bundles with no central communicating structure, and type 3 is characterized by no mucosal communication or smooth muscle boundary and a significantly distorted tissue organization.^27^ The most frequent subtype was type 1 (6/12 patients), followed by type 2 (4 patients), and type 3 (2 patients).^27^ The characteristic feature of type 1 GHIP may represent the endoscopic feature of an opening in the center to the surface with mucus outflow, which is an occasional finding of GHIPs (15 tumors). According to this classification, two tumors of the present series were classified as type 2 (Patients 2 and 3) because no communication between the submucosal lesion and the surface mucosa was noted, whereas the remaining tumor was classified as type 3 (Patient 1). As described above, differentiating GHIPS from other SMTs of the stomach, including those of the submucosal heterotopic gastric mucosa and gastritis cystica profunda, might be difficult ^27^; thus, additional clinicopathological studies are needed to clarify the classification of the SMTs of the stomach. Of importance, three of six patients with a type 1 GHIP were shown to coexist with an adenocarcinoma within the lesion of a GHIP, and a hyperplasia–dysplasia–carcinoma sequence was also observed within the lesion.^27^ Thus, a type 1 GHIP may have malignant potential, and the reason for this may be that the central communicating structure allowed a continuous exposure of luminal carcinogen and mechanical stress to the submucosal glandular cells of a GHIP.^27^ The present study consisted of type 2 and type 3 GHIPs, and no carcinomatous component was noted within the lesion. Moreover, gene analysis demonstrated no significant mutations in any of the three tumors.

To date, immunohistochemical analyses of the epithelial cells composed of the submucosal lesion have been performed in eight GHIP tumors, including our series (adenocarcinoma arising: one case).^15^, ^16^, ^17^, ^22^, ^25^ All the tumors had MUC5AC‐ and MUC6‐positive components, which suggested that all the GHIPs had components of the foveolar epithelium and pyloric gland or mucous neck cells. In addition, pepsinogen‐I and H^+^/K^+^ ATPase‐positive cells were noted in four tumors, which demonstrated the presence of fundic gland components. In the present series, two tumors of the type 2 GHIP had an MUC5AC‐positive foveolar epithelium component at the center of the cystically dilated glands and MUC6 or pepsinogen‐I and H^+^/K^+^ ATPase‐positive cells at the periphery of the cystically dilated glands, which suggested the presence of a normal gastric mucosal architecture. Although the pathogenesis of GHIPs has not yet been clarified, Kim *et al.* speculated that repeated mucosal inflammation caused a break in the muscularis mucosae, which permitted a downward herniation and submucosal trapping of the mucosal glands.^11^

In conclusion, we reported on three patients with a GHIP and reviewed the clinicopathological and endoscopic features of this lesion. Although approximately three fourths of GHIPs show an SMT‐like feature, endoscopic features, including EUS, are not characteristic. An endoscopic diagnosis of a GHIP may be difficult; therefore, complete endoscopic resection may be needed for the pathological diagnosis. Additional studies are required to clarify the classification of SMTs of the stomach and to verify the malignant potential of GHIPs.

## CONFLICTS OF INTEREST

None.

## Supporting information

Supplemental table for Table 3
