# Supplementary material for: Gastric hamartomatous inverted polyp: Report of three cases with a review of the endoscopic and clinicopathological features
Source: DEN Open. 2023 Jan 4;3(1):e198. doi: 10.1002/deo2.198 (PMC9812834; doi:10.1002/deo2.198)
Supplement: Supplementary file 1 — Supplemental table for Table 3 [file DEO2-3-e198-s001.docx]

**Supporting information**

Supplemental table for Table 3. Summary of the clinicopathological and endoscopic features of the previously reported gastric hamartomatous inverted polyp.

| **Patient No.** | **Polyp No.** | **Reference** | **Age** | **Sex** | **Site** | **SMT / polyp type** | **Size (mm)** | **Endoscopic findings** | |
| --- | --- | --- | --- | --- | --- | --- | --- | --- | --- |
|  |  |  |  |  |  |  |  | **Features of the surface** | **Aperture to the surface** |
| 1 | 1 | 2 | 47 | F | Fundus | Polyp | 13 | Normal | ○ |
| 2 | 2 | 3 | 66 | F | Fundus | Polyp | 15 | Normal | × |
| 3 | 3 | 4 | 79 | M | Cardia | SMT | 25 | Normal | ○ |
| 4 | 4 | 5 | 41 | F | Fundus | Polyp | 23 | Normal | × |
| 5 | 5 | 6 | 69 | M | Body | SMT | 10 | NA | ○ |
| 6 | 6 |  | 58 | M | Cardia | SMT | 26 | NA | × |
| 7 | 7 |  | 34 | F | Body | SMT | 30 | NA | ○ |
| 8 | 8 |  | 81 | M | Fundus | SMT | 5 | NA | × |
|  | 9 |  |  |  | Body | SMT | 7 | NA | × |
| 9 | 10 | 7 | 43 | F | Body | SMT | 28 | Erosive and depression | × |
| 10 | 11 | 8 | 54 | M | Antrum | Polyp | 45 | Normal | ○ |
| 11 | 12 | 9 | 59 | M | Body | SMT | NA | Focal discoloration | × |
| 12 | 13 | 10 | 37 | M | Fundus | SMT | >20 | Erosion and depression | × |
| 13 | 14 | 11 | 40 | F | Body | SMT | 3.5 | Round superficial nodular changes | × |
| 14 | 15 | 12 | 77 | M | Body | SMT | 5 | NA | × |
| 15 | 16 | 13 | 31 | F | Body | SMT | 30 | Reddish | × |
| 16 | 17 |  | 65 | M | Body | SMT | 40 | Reddish and Erosive | × |
| 17 | 18 | 14 | 23 | F | Fundus | SMT | 18 | Normal | ○ |
| 18 | 19 | 15 | 70 | M | Body | Polyp | 10 | Normal | × |
| 19 | 20 | 16 | 66 | M | Body | SMT | 15 | Normal | × |
| 20 | 21 | 17 | 70 | M | Body | SMT | 16 | Erosion and hemorrhaging | × |
| 21 | 22 | 18 | 55 | F | Body | SMT | 15 | Depression | × |
| 22 | 23 | 19 | 58 | F | Body | SMT | 25 | NA | ○ |
| 23 | 24 | 20 | 64 | F | Body | Polyp | 13 | NA | × |
| 24 | 25 | 21 | 37 | F | Fundus | SMT | 10 | Depression | ○ |
| 25 | 26 | 22 | 54 | M | Body | SMT | 35 | NA | ○ |
| 26 | 27 | 23 | 46 | M | Body | SMT | 33 | Depression | NA |
| 27 | 28 | 24 | 75 | F | Body | Polyp | NA | Dilated vessels | ○ |
| 28 | 29 | 25 | 60 | M | Body | SMT | 35 | Depression | × |
| 29 | 30 | 26 | 38 | F | Antrum | SMT | 4 | Depression | ○ |
| 30 | 31 | 27 | 72 | F | Fundus | SMT  (Type1) | 20 | Hyperemic elevation | ○ |
| 31 | 32 |  | 50 | M | Antrum | SMT  (Type1) | 15 | Hyperemic elevation | ○ |
| 32 | 33 |  | 51 | M | Body | SMT  (Type1) | 12 | Erosion | ○ |
| 33 | 34 |  | 51 | M | Body | SMT  (Type1) | 14 | Depression | ○ |
| 34 | 35 |  | 50 | F | Body | SMT  (Type1) | 10 | Orifice with mucin | ○ |
| 35 | 36 |  | 34 | F | Body | SMT  (Type1) | 13 | Depression | ○ |
| 36 | 37 |  | 69 | M | Body | NA (Type2) | 10 | NA | × |
| 37 | 38 |  | 77 | M | Body | SMT  (Type2) | 25 | Erosion | × |
| 38 | 39 |  | 70 | M | Body | SMT  (Type2) | 13 | Normal | × |
| 39 | 40 |  | 58 | M | Body | SMT  (Type2) | 10 | Normal | × |
| 40 | 41 |  | 54 | F | Body | Polyp  (Type3) | 15 | Normal | × |
| 41 | 42 |  | 35 | M | Body | Polyp  (Type3) | 22 | Normal | × |

F, Female; M, Male; NA, Not available; SMT, Submucosal tumor
